# Supplementary material for: Ultrasonographic findings in patients with abdominal symptoms or trauma presenting to an emergency room in rural Tanzania
Source: PLoS One. 2022 Jun 2;17(6):e0269344. doi: 10.1371/journal.pone.0269344 (PMC9162326; doi:10.1371/journal.pone.0269344)
Supplement: Attachment — Submitted filename: Response to Reviewers.pdf [file pone.0269344.s001.pdf]

Point by Point Reply

Journal Requirements:

When submitting your revision, we need you to address these additional requirements.

1. Please ensure that your manuscript meets PLOS ONE's style requirements, including those for file naming. The PLOS ONE style templates can be found at https://journals.plos.org/plosone/s/file?id=wjVg/PLOSOne_formatting_sample_main_body.pdf and https://journals.plos.org/plosone/s/file?id=ba62/PLOSOne_formatting_sample_title_authors_affiliations.pdf

We thank the reviewer for this comment. The manuscript was changed to meet the stated style requirements as shown in the links above.

2. Your ethics statement should only appear in the Methods section of your manuscript. If your ethics statement is written in any section besides the Methods, please delete it from any other section.

- We thank the reviewer for this comment. The ethics statement at the end of the manuscript was deleted.

3. Please review your reference list to ensure that it is complete and correct. If you have cited papers that have been retracted, please include the rationale for doing so in the manuscript text, or remove these references and replace them with relevant current references. Any changes to the reference list should be mentioned in the rebuttal letter that accompanies your revised manuscript. If you need to cite a retracted article, indicate the article's retracted status in the References list and also include a citation and full reference for the retraction notice.

- The references were revised. We added Ref 29 on page 7 in the methods section.

Additional Editor Comments:

Title
The word disease is not suitable as it excludes individuals with normal findings. Also, some findings are not unique diseases but a reflection for an existing disease.

-We thank the reviewer for this valuable comment. “Diseases detected with sonography in patients with abdominal symptoms or trauma presenting to an emergency room in rural Tanzania”
was changed to “Ultrasonographic findings in patients with abdominal symptoms or trauma presenting to an emergency room in rural Tanzania”

Abstract

Please write the aim of the study included in background subsection Introduction (not mentioned)

- We thank the reviewer for this comment. The aim of the study was included in the Background section of the abstract: “Frequencies of ultrasonographic findings and diagnoses in emergency departments in sub-Saharan Africa are unknown. This study aimed to describe the frequencies of different sonographic findings and diagnosis found in patients with abdominal symptoms or trauma presenting to a rural referral hospital in Tanzania.”
- The sentence “All ultrasound examinations were done by board-certified clinicians in emergency point of care ultrasound.” Was deleted to reduce the wordcount below 300 words.

Please change the subtitle Background (that is next to abstract) to introduction.

- Background was changed to introduction in the text.

Ethics consideration

All studies conducted on human have to be conducted in accordance with Helsinki standards (please state this in ethics statement)

- “The study was conducted in accordance with the Declaration of Helsinki” was included in the ethic statement.

Table 1

The definition of adults to be less than 14 years is not correct (less than 18 years), please correct

- We thank the reviewer for this comment. Since <14 years is the cut off at St. Francis Referral Hospital for being a child, we devided the patients into ≥14 years (adolescents and adults) and <14 years.

Table 3
How did you diagnose pneumothorax by ultrasound

- We thank the reviewer for this comment. A description and reference defining the diagnosis of pneumothorax was added to the study procedures within the methods section: "Diagnostic criteria for pneumothorax was absence of lung sliding, comet tail artefacts, and sea-shore sign in M-mode, or demonstration of lung point." On page 7. We added also Ref 29.
- A description of the diagnosis of pneumothorax was also added to the footnotes of Table 3.

The footnotes mentioned below table 3 are not related to it, they are belonging to table 2, so please put them below table 2.

- -We checked and revised the footnotes of all tables, which are correct now.

Table 4
You mentioned footnotes below it, however, no such notes in the table itself, correct.

- We apologize for this mistake. We removed the old footnotes, but added new footnotes a and b to define pneumonia and lung edema

In references
For websites, please write the links and accessed date.
Also revise other references to add page range and complete details of cited books, if any.

- The reference 2 "WHO. Injuries and violence: the facts 2014. World Health Organization Geneva; 2014.»
  was replaced by «World Health Organization. Global Status Report on Road Safety 2009: time for action. Geneva:World Health Organization; 2009. Available from: http://apps.who.int/iris/handle/10665/78256. »

Ifakara Branch
Off Mlabani Passage
P.o. Box 53 Ifakara
Phone: +255232931572

Dar es Salaam Office
Plot 463, Kiko Avenue, Mikocheni
P.o. Box 78,373 Dar es Salaam
Phone: +255222774756

Bagamoyo Branch
Inside District Hospital
P.o. Box 74 Bagamoyo
Phone: +255232625164

- The reference 3 “Clark M, Spry E, Daoh K, Baion D, Skordis-Worrall J. Reductions in inpatient mortality following interventions to improve emergency hospital care in Freetown, Sierra Leone. 2012.»
  was corrected with the journal and page numbers: «Clark M, Spry E, Daoh K, Baion D, Skordis-Worrall J. Reductions in inpatient mortality following interventions to improve emergency hospital care in Freetown, Sierra Leone. PLoS One. 2012;7(9):e41458.»
- Page numbers were added to the reference 25 «Ngome O, Rohacek M. Point-of-Care Ultrasound: A Useful Diagnostic Tool in Africa. Praxis. 2020;109(8):608-14»
- Page numbers wree added to the reference 27 «Rohacek M, Hatz C, Weisser M. Emergency Department in the St. Francis Referral Hospital, Ifakara, Tanzania. Praxis. 2017;106(12):651-5.»
- Reference 32 was corrected to «WHO, UNICEF, UNFPA, World Bank Group, UN Population Division. Trends in maternal mortality: 1990 to 2015. Available from: http://apps.who.int/iris/bitstream/10665/194254/1/9789241565141_eng.pdf?ua=1.»

Reference 48 was changed to the book: «American College of Surgeons. 10th Edition of the Advanced Trauma Life Support® (ATLS®) Student Course Manual. Chicago: American College of Surgeons; 2018.»
